# Uncovering low-level mosaicism in human embryonic stem cells using high throughput single cell shallow sequencing

**DOI:** 10.1038/s41598-019-51314-6

**Published:** 2019-10-16

**Authors:** Alexander Keller, Laurentijn Tilleman, Dominika Dziedzicka, Filippo Zambelli, Karen Sermon, Filip Van Nieuwerburgh, Claudia Spits, Mieke Geens

**Affiliations:** 1 Research Group Reproduction and Genetics, Vrije Universiteit Brussel, Laarbeeklaan 103, 1090 Brussels, Belgium; 2 Laboratory of Pharmaceutical Biotechnology, Ghent University, Ottergemsesteenweg 460, 9000 Ghent, Belgium; 3 Clínica EUGIN, Travessera de les Corts 322, Barcelona, 08029 Spain

**Keywords:** Genomic instability, DNA sequencing, Embryonic stem cells

## Abstract

Human pluripotent stem cells (hPSCs) have significant levels of low-grade genetic mosaicism, which commonly used techniques fail to detect in bulk DNA. These copy number variations remain a hurdle for the clinical translation of hPSC, as their effect *in vivo* ranges from unknown to dangerous, and the ability to detect them will be necessary as the field advances. As such there is need for techniques which can efficiently analyse genetic content in single cells with higher throughput and lower costs. We report here on the use of the Fluidigm C1 single cell WGA platform in combination with shallow whole genome sequencing to analyse the genetic content of single hPSCs. From a hPSC line carrying an isochromosome 20, 56 single cells were analysed and found to carry a total of 50 aberrations, across 23% of cells, which could not be detected by bulk analysis. Aberrations were predominantly segmental gains, with a fewer number of segmental losses and aneuploidies. Interestingly, 40% of the breakpoints seen here correspond to known DNA fragile sites. Our results therefore demonstrate the feasibility of single cell shallow sequencing of hPSC and further expand upon the biological importance and frequency of single cell mosaicism in hPSC.

## Introduction

Human pluripotent stem cell (hPSC) cultures are known to readily acquire a significant number or recurrent copy number variations (CNVs) across lines worldwide^1,2^. While these CNVs eventually take over the entire culture, many are found to be midway through that process as a mosaic population^3,4^. Taapken and colleagues^3^, for example, analysed over 1700 hPSC cultures from 29 laboratories worldwide and found commonly recurrent gains on chromosomes 8, 12, 12p, 17, i(20)(q10) and X to often be mosaic in the range of 5 to 95% across a significant portion of their samples. Strikingly, they also found that 40% of all CNVs observed were not part of these recurrent aberrations and were randomly distributed throughout the genome. The most commonly recurring CNVs are acquired easily and often, but globally, CNVs have been described on every chromosome, indicating that genomic instability is not limited to a select few regions within the genome and is inherent in hPSC under current culture conditions^3–6^. Importantly, these early studies relied predominantly on low resolution techniques such as G-banding, which fail to detect smaller segmental aberrations and likely underestimate the true extent of genetic heterogeneity of hPSC. More recent work using higher resolution single cell array-based comparative genomic hybridization (aCGH) confirmed and expanded upon these early findings, showing that hPSCs cultures carry a low but significant number of karyotypically aberrant cells which are missed in bulk analysis and fall below the resolution of traditional banding techniques^7^. Cells with recurrent aberrations acquire a significant survival advantage and takeover quickly (as few as 10 passages)^8–10^, while non-recurrent aberrations, which do not provide an advantage to the cell, remain in drift and will not be selected for over prolonged culture, but contribute nonetheless to the highly mosaic character of hPSC.

These low-level events may be of significant importance as the field transitions into the clinic, given that many of the CNVs commonly found in hPSC are also present in several cancer types^10–12^ and can interfere with stem cell differentiation^13^. Furthermore, while non-recurrent aberrations may have no significant impact on hPSC, their effect on differentiated cells remains largely unknown. Given that a high number of cells are necessary for therapeutic applications, large-scale hPSC production will be essential for their successful application in regenerative medicine. The number of cells required for effective treatment varies according to the therapeutic goal, but is expected to range between millions and billions of cells^14^. Also, for biomedical research purposes, such as disease modelling and drug discovery, large numbers of cells will be required^15^. Such high cell counts increase the risk of low-level mosaics passing unnoticed, and potentially being transplanted into patients or interfering with experimental results.

In order to evaluate the mosaic nature of a population of cells, it will be necessary to develop more sensitive methodologies for low level CNV detection. Currently, commonly used methods to evaluate genetic content, such as chromosome banding, aCGH, FISH and PCR-based detection (qPCR and ddPCR) are effective but suffer from a variety of shortcomings linked to inherent limitations of the given technique^2^. PCR-based detection is limited to the identification of CNVs present in 5–10% of cells and both PCR-based techniques and FISH are limited by the need for a specific target for detection. Banding techniques have a limited resolution and are restricted in the types of aberrations they are able to identify. Analysis of the genetic content of single cells by aCGH or shallow whole genome sequencing (sWGS) could overcome this shortcoming and allow for the detection of unknown variants within the cell line. However, identifying these mutations in single cells requires the manual extraction and whole genome amplification (WGA) of individual cells, which can be both time consuming and cost prohibitive for large cell numbers, limiting the detection of low-level mosaic mutants.

Microfluidic devices, such as the Fluidigm C1 single cell WGA platform, could offer an alternative to currently available techniques and can be used in conjunction with sWGS for high throughput single cell analysis. The C1 platform utilizes an integrated fluidic circuit (IFC) to automatically sort single cells into individual reaction chambers where nanolitre volume amplification can occur. This allows for a reduction in time input per sample, while dramatically decreasing the required reagent input and cost compared to traditional benchtop techniques. Furthermore, microfluidic environments can reduce the risk of sample contamination and increase reaction efficiency, making them ideally suited for single cell analysis.

We report here the sWGS results of 56 single human embryonic stem cells (hESC), the largest populations, to our knowledge, of sWGS hESC in the literature. We also demonstrate that the combination of sWGS with WGA via the Fluidigm C1^16^ is a viable platform for the identification of large CNVs at the single cell level. A cohort of the single cells carried large aberrations not found in the analysis of the bulk DNA, including both aneuploidies and segmental aberrations. These findings are in agreement with other published works on the presence of low-level chromosomal abnormalities in hPSC. Therefore, this approach fills a gap in currently available techniques for the analysis of the genetic content in single cells.

## Results

### Single cell CNV detection strategy

**Figure 1:**
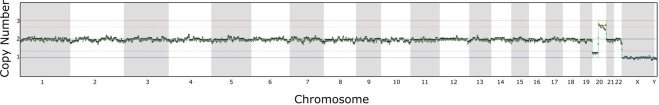
Shallow whole genome sequencing of VUB02_iso20: The genetic profile of VUB02_iso as seen by shallow whole genome sequencing at a bin size of 0.5 Mb. The line displays a balanced genetic content aside from the isochromosome 20.

We used the Fluidigm C1 to automate the separation and DNA amplification of individual cells for sWGS. To validate low level CNV detection, we utilized a hESC line which carried known abnormalities. We identified a subline of VUB02, VUB02_iso20, carrying an isochromosome 20 with a loss of the p-arm and a gain of the q-arm (46,XY,i(20q11)). VUB02_iso20 was chosen as it would give a representation of both gains and losses, as well as different sized aberrations for the p and q arms. This aberration was initially detected by aCGH of a bulk of cells, and later confirmed by sWGS (Fig. 1). The isochromosome 20 failed to reach copy 1 for the p and copy 3 for the q arm after sWGS, instead the predicted copy numbers were 1.3 and 2.74 respectively. This strongly suggested that the population of cells was mosaic for the isochromosome.

### Cell capture and amplification

**Figure 2:**
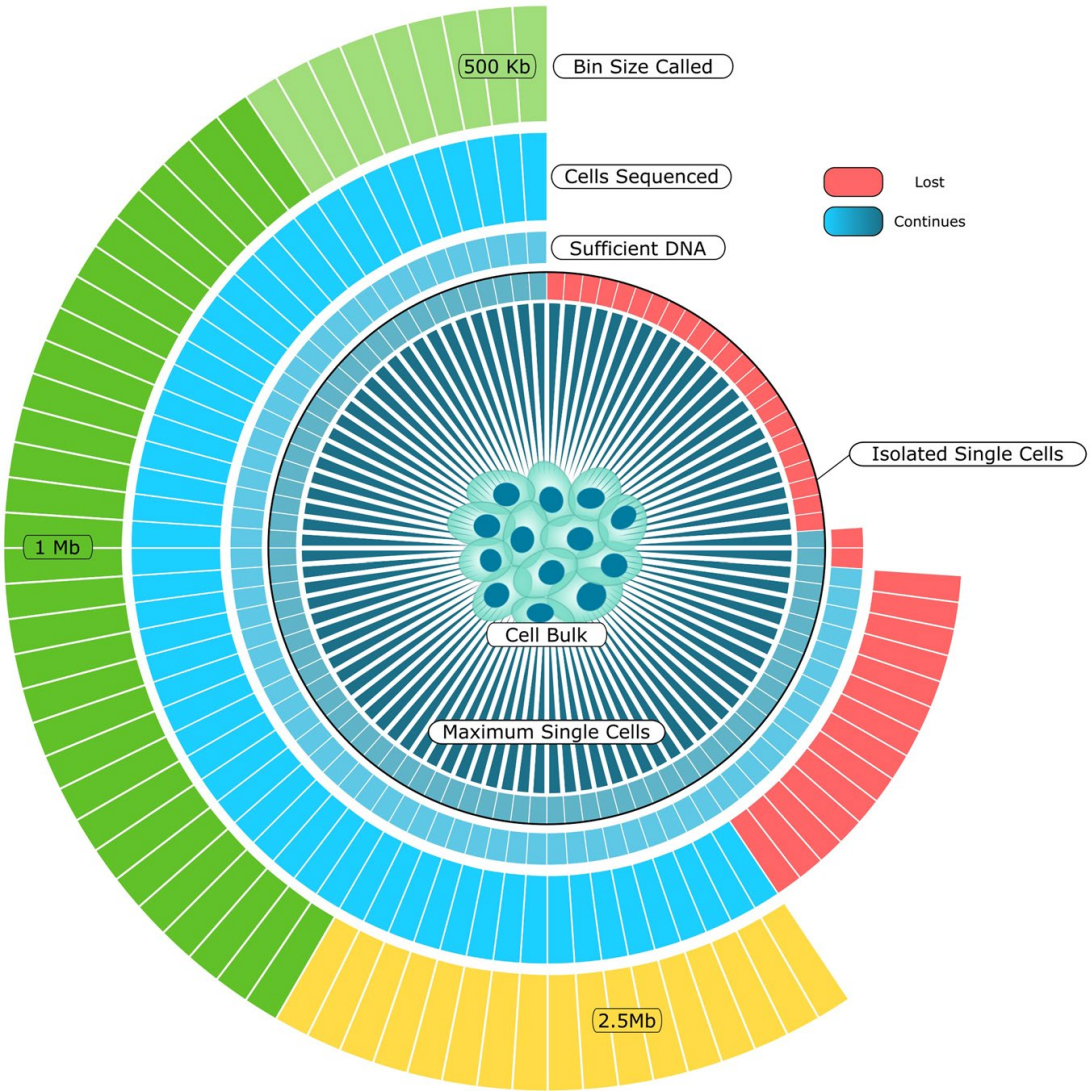
Outcome of individual steps from cell isolation to sequencing: Schematic overview of the progression of individual cells from isolation through to final analysis. Red blocks indicate a loss of a cell at a given step in the protocol either by failure to isolate, or a failed quality check, with cell isolation and failed sequencing representing the points of greatest overall loss in the protocol.

The Fluidigm IFC’s can capture up to 96 cells on cell capture sites located on the chip, with multiple chip sizes available depending on the cell size needed. hPSC have a standard size distribution between 10–18 µm as measured by the Tali™ Image-based Cytometer, as such a 10–17 µm IFC was used. Only capture sites with a single cell and a uniform round morphology were taken for sequencing. A majority of capture sites held single cells (74%), 14.5% carried multiple cells, 9.5% had captured no cells and 2% had cells within the reaction region but not directly on the capture site, which does not affect their amplification (see Supplemental Fig. S1). From the initial bulk population, 73 cells were captured and available for amplification (Fig. 2).

There are currently numerous WGA systems available which promise balanced amplification of the genome, a prerequisite for a correct calling of CNVs. For this study SurePlex chemistry was used as it was recently demonstrated to be best suited for WGA with the goal of evaluating CNVs^17^. The yield after amplification was 0.79 ± 0.17 ng/µl. Of the 73 single cells captured and amplified, 69 yielded sufficient DNA for sequencing (Fig. 2).

### Identification of unique mega-base scale aberrations

After sequencing, reads were put into contiguous bins from which 5 bins were clustered into larger segments. Total read counts across segments was then used to determine local copy number (described below). Analysis was performed at three bin sizes, 0.5 Mb, 1 Mb and 2.5 Mb, after which cells were qualitatively categorized into the most appropriate bin size for final analysis based on the distribution of bins throughout the sample. Higher quality samples had tighter grouping of bins within ±0.25 of integer copy numbers (1, 2 and 3) allowing for analysis at smaller bin sizes and therefore increased resolution and more precise breakpoint localization. Of the 69 samples sequenced, 56 yielded sequencing results of sufficient quality for analysis, 9/56 being analysed at 0.5 Mb, 31/56 at 1 Mb and 16/56 at 2.5 Mb (Fig. 2).

**Figure 3:**
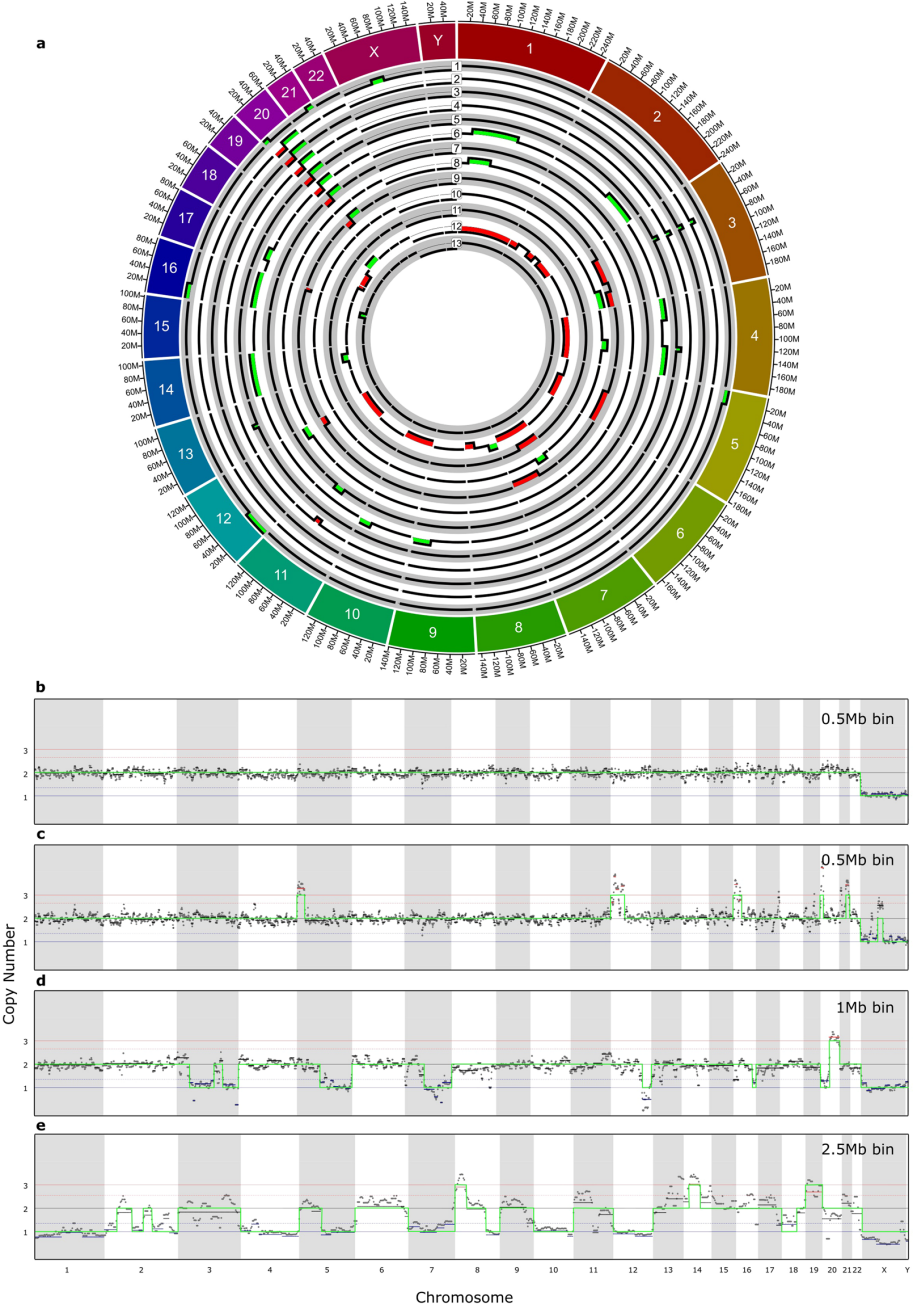
Overview of *de novo* CNVs seen in single cells of VUB02_iso20: (**a**) An overview of single cells which carried additional CNVs not seen in bulk analysis (**b**–**e**) Examples of cells analysed at different bin sizes. (**b**) A single cell analysed at a bin size of 0.5 Mb with normal genetic content. (**c**) Cell number 1 from (**a**) analysed at 0.5 Mb (d) Cell number 9 from (**a**) analysed at 1 Mb (**e**) Cell number 12 from (**a**) analysed at 2.5 Mb.

Only 40/56 (71%) of the single cells carried the isochromosome, confirming the data on the bulk cells suggesting that the culture was mosaic. In three cells, either the deletion of the p arm (1/3) or the duplication of the q arm (2/3) was not called. A significant number of CNVs were found in addition to the isochromosome (Table 1), 23% (13/56) of cells carried at least one additional CNV of ≥8 Mb (Fig. 3). Of the total 50 CNVs detected, a majority were sub-chromosomal gains (28/50), with a smaller number of sub-chromosomal losses (14/50). Gains by trisomy (3/50) and losses by monosomy (5/50) were relatively few. Of note were 4 cells (Fig. 3a #1, 6, 9 and 12, c–e) carrying four or more aberrations as part of complex karyotypes, one of which (Fig. 3a #12, e) could be considered a chaotic genome, with 14 aberrations. Together they represent a disproportionate share (36/50) of the total number of aberrations detected across all cells, not including the isochromosome.

**Table 1:** CNV breakpoints and fragile sites.

Cell	Chromosome	Start	End	Size	Fragile Site
1	5	1	26 Mb	26 Mb	FRA5E
	12	1	48 Mb	48 Mb	FRA12A
	16	1	29 Mb	29 Mb	FRA6E
	20	1	11.5 Mb	11.5 Mb	FRA20B
	21	33 Mb	46.5 Mb	13.5 Mb	
	X	57.5 Mb	81.5 Mb	24 Mb	
2	3	60.5 Mb	69.5 Mb	9 Mb	
3	3	60.5 Mb	69.5 Mb	9 Mb	FRA3B/none
4	3	60.5 Mb	69.5 Mb	9 Mb	FRA3B/none
	11	68 Mb	82 Mb	14 Mb	none/FRA11EH
5	3	60.5 Mb	69.5 Mb	9 Mb	FRA3B/none
	4	119.5 Mb	129.5 Mb	10 Mb	
	13	50 Mb	59 Mb	9 Mb	none/FRA13b
6	1	35.5 Mb	150.5 Mb	115 Mb	none/FRA1F
	2	158.5 Mb	242 Mb(T)	84 Mb	
	4	1	54.5 Mb	54.5 Mb	FRA4b
	4	118 Mb	190 Mb(T)	72 Mb	
	9	95 Mb	138.5 Mb(T)	43.5 Mb	
	10	104 Mb	134 Mb(T)	30 Mb	FRA10A
	14	1	107 Mb	107 Mb	
	16	1	90 Mb	90 Mb	
	17	27.5 Mb	58.5 Mb	31 Mb	none/FRA17B
7	11	68.5 Mb	95.5 Mb	27 Mb	FRA11EH/none
8	1	30 Mb	94 Mb	64 Mb	FRA1D/FRA1M
	12	100 Mb	133 Mb	33 Mb	
9	3	38.5 Mb	120.5 Mb	82 Mb	
	3	149.5 Mb	198.5(T)	49 Mb	FRA3D
	5	79 Mb	181.5 Mb(T)	102.5 Mb	
	7	69 Mb	159.5 Mb(T)	90 Mb	FRA7J
	12	106 Mb	133.5 Mb(T)	27.5 Mb	
	16	81 Mb	90 Mb(T)	9 Mb	FRA16D
10	3	137.5 Mb	198.5 Mb(T)	61 Mb	
	4	105 Mb	142 Mb	37 Mb	
	7	16.5 Mb	52 Mb	35.5 Mb	
11	7	1	87 Mb	87 Mb	
12	1	1	249 Mb	249 Mb	
	2	1	40 Mb	40 Mb	
	2	98 Mb	134.5 Mb	36.5 Mb	FRA2A/FRA2S-2F
	2	164 Mb	242 Mb (T)	78 Mb	
	4	1	190 Mb	190 Mb	
	5	75.5 Mb	181.5 Mb(T)	106 Mb	
	7	1	159.5 Mb	159.5 Mb	
	8	1	39 Mb	39 Mb	
	8	103 Mb	145 Mb(T)	42 Mb	FRA8A
	10	1	134 Mb	134 Mb	
	12	1	133 Mb(T)	133 Mb	
	14	38.5 Mb	73 Mb	34.5 Mb	
	18	1	54.5 Mb	54.5 Mb	FRA18B
	19	1	58.5 Mb	58.5 Mb	
13	16	31.5 Mb	55 Mb	23.5 Mb	

An overview of CNV calls, breakpoints, sizes and fragile sites across all 13 single cells carrying additional CNVS. T denotes the endpoint is terminal.

Apart from the isochromosome 20, an 9 Mb gain on chromosome 3 in four cells and the loss of the q-arm of chromosome 5 in two cells were the only aberrations found at the same position in more than one cell. All other detected CNVs were unique, though two non-identical sub-chromosomal gains clustered in the same region of chromosome 1 (Fig. 3a # 6, 8). None were detectable in the original bulk population of cells. Of note, we found a gain and a loss of a similar size at the telomere of chromosome 12q in cells 8 and 9 respectively.

Given the high number of *de novo* CNVs found across the single cells, we cross referenced the breakpoint regions to known fragile sites in the human genome using HumanCFS, a database of experimentally validated fragile regions (https://webs.iiitd.edu.in/raghava/humcfs/index.html). Of the 60 breakpoints observed, 24 (40%) fell within known fragile sites (Table 1), with a further 9 (not shown) falling within 2.5 Mb (maximally one additional bin) of known fragile sites. The proximal breakpoint in the 9 Mb gain on chromosome 3 (cells 2, 3, 4 and 5) falls within FRA3B, which is classified as highly fragile and may account for its frequency.

## Discussion

In this report, we describe a method to detect CNVs, both aneuploidies and segmental aberrations, in a large number of single cells simultaneously. To our knowledge, this is the first report on shallow sequencing of such a population of single hESC. Our results demonstrate that combining the Fluidigm C1 platform with sWGS can be used to detect unique mega-base scale aberrations as well as determine with high sensitivity the level at which a given aberration is mosaic within a population of cells. Furthermore, this methodology could be used as an indicator of the mosaic nature of a given cell line and could be used as a tool to evaluate different culture systems effect on genome stability. Beyond using this technique to evaluate the genetic content in hPSC, a variety of other fields could benefit from the ability to parse out the genetic content of a complex population of cells. Heterogeneity continues to be an obstacle for the effective understanding of tumour makeup for example, and our understanding of heterogeneity in humans continues to be challenged as an increasing number of cell types in healthy individuals are shown to be mosaic and carry a variety of CNVs^18–21^. Here we analysed 56 single cells simultaneously and identified 13 cells with 50 unique aberrations not found in the bulk analysis of the original cell population. Though a majority of the aberrations are random in nature, a small number do correspond to known recurring abnormalities in hPSC lines worldwide. Specifically, a segmental gain in chromosome 12, trisomy 14, 16 and a loss of the q-arm of chromosome 7 are observed and consistent with previously reported work^1,2^. Interestingly, the genetically reciprocal gain and loss on chromosome 12, though only present in a single pair, may have been the result of an error in segregation during mitosis in the past, which has since remained in drift within the total population. Perhaps most interestingly is the presence of a cell with a highly complex, often referred to as chaotic, genome found in cell 12, and 3 other cells with complex combinations of aberrations. The mechanisms leading to such events is not yet fully understood but may result from stress placed on the cell and is often seen as a hallmark of cancer^22,23^. Furthermore, the frequency of CNVs found to have breakpoints at common fragile sites is striking, as genetic instability is another hallmark of cancer. While the instability seen in hPSC is now well established, this nonetheless further demonstrates their high susceptibility to DNA damage, especially at fragile sites. It is possible that in our experiments the manipulation of the cells led to increased stress at these fragile sites, however this is unlikely to have had an effect on the final CNV calls. The presence of DNA breaks would not change the total genetic content found within each capture site, and a segmental gain or loss would have been the result of an event in an ancestral cell.

The use of different WGA techniques may also play a role in the correct detection of CNVs in single cells, and the rate of false positive or negative calls. Sure Plex has long been used as the gold standard for CNV detection in aCGH analysis for preimplantation genetic testing and has been shown to be well suited to CNV detection in single cells^24–26^. Recently, Deleye and colleagues^17^ demonstrated that Sureplex was better suited for the detection of CNVs than MALBAC WGA, with significantly fewer, though not zero, false positives. As such, while minimized both through the selection of the WGA method, strict quality control and threshold setting, there does remain the possibility of false positive calls within the findings presented here. Ruling out whether each individual CNV is a false positive is not possible however, due to the often-unique nature of each CNV, or their low frequency and the detection limit of available techniques, further discussed below.

While a singular cell line is not representative of all hPSC lines currently in use, our results nonetheless highlight the limitations of bulk analysis. It is striking to find that 23% of the cells carry at least one aberration in addition to the isochromosome 20, especially given the relatively small population analysed in comparison to an entire culture of cells. Furthermore, the appearance of the isochromosome 20 was a recent event in the history of the cell line, having not been detected in bulk analysis only 5 passages earlier, indicating that even after this bottleneck event, CNVs develop quickly within a culture.

Given the large number of CNVs seen here and the need for perhaps billions of cells in therapeutic applications, it becomes clear that more work is needed to develop conditions which reduce genomic instability in hPSC, as current methods may lead to increased stress on the cells^27^. Many CNVs found in hPSC, some of which are described here, could potentially result in dangerous complications in therapeutic applications. For example, the gain at 20q11.21 is highly recurrent^1,2,12^ and has extensive links to cancer^28,29^. Furthermore, both the gain of 20q11.21 and chromosome 12 (seen here) have demonstrated features of neoplastic progression in hPSC^30^, with gains of chromosome 12 also showing increased tumorigenicity^31^. Loss of chromosome 7q has also been linked to myelodysplastic syndrome and acute myeloid leukaemia^32^. Furthermore, the many other gains and losses, while not all described, may still result in altered gene expression patterns in those cells, with unknown consequences. As an example, the 9 Mb gain on chromosome 3 seen here in 4 cells has links to cancer^33^ and passed by bulk analysis undetected. While low level mosaics have been highlighted in previous work, the results here further emphasize their frequency and importance.

Single cell genetic analysis by aCGH has slowly been replaced by sWGS, even so, an increase in throughput for either technique necessitates a significant increase in cost, limiting their application. Microfluidic devices bypass this limitation with their use of nanolitre volumes of reagents, significantly reducing the cost of the amplification and sequencing required for single cells, as well as increasing throughput. This technique fills a gap in the current literature for the detection of low level CNVs in single cells, as current methods are limited to high sensitivity bulk analysis (aCGH, sWGS), and targeted lower throughput with limited sensitivity (FISH). As the genetic status of hPSC is increasingly coming into question the closer they move to the clinic, better techniques will be required to accurately evaluate their safety.

This technique does have shortcomings however, most importantly is its relatively low resolution compared to bulk analysis. While many recurring chromosomal abnormalities are aneuploidies, several highly recurrent aberrations have a minimally amplified region that is too small for detection. For example, the gain at 20q11.21, which has a minimal reported amplicon of 1 Mb or less^10^, would have been below the strict thresholds set here. As such, the breakpoints identified here are also limited in their resolution. Furthermore, the Fluidigm C1 is relatively limited in its output, with a maximum of 96 cells per run. Other microfluidic systems, especially those which generate emulsified droplets, can generate tens of thousands of encapsulated samples. While the overall price of single cell experiments has shrunk as a result of these devices, one of the main costs still remains the library preparation. While automated massive single cell barcoding systems, which utilize hydrogels with unique barcodes to generate vast libraries have existed for RNAseq for some time^34^, only very recently have microfluidic systems enabled this for DNA with the recent release of the 10X genomic system for single cell CNV detection. Still, the Fluidigm C1 platform allows for detection of karyotypic abnormalities in hPSC at single cell levels with a moderately low cost and opens the possibility of answering novel questions related to single cell heterogeneity. Lastly, the inherent challenge of validating any CNV detected within a single cell remains. Jacobs and colleagues^7^ attempted to overcome this by including single cell clones and FISH in their analysis. While they identified low-level CNVs not seen in the bulk, they did not find back the same CNVs as in their single cells. As such, they confirmed the mosaic nature of the cells, but the unique nature of individual CNVs precludes the possibility of validation by a second technique.

In conclusion, we demonstrate that the Fluidigm C1 is a viable platform for the identification of CNVs in single hPSC. The findings we present here also demonstrate the biological importance of investigating the mosaic nature in hPSC in large populations of cells, and that further work is needed to demonstrate the true heterogenous nature of hPSC.

## Materials and Methods

### Cell culture

hESC line VUB02 was derived and characterized as previously described^35^. Cells were routinely cultured at 37 °C at 5% CO2 on dishes coated with Laminin521® at 10 µg/cm^2^ (Biolamina) in Nutristem® XF medium (Biological Industries) supplemented with 0.1% penicillin/streptomycin (Thermo Fisher Scientific). Cells were passaged as single cells using TrypLE™ Express (Thermo Fisher Scientific) when 80–90% confluent in a ratio between 1:10 and 1:40. Initial karyotype was determined by aCGH (4 × 44 K, Agilent Technologies) as previously described^7^.

### Single cell whole genome amplification

Single cell WGA was performed on the Fluidigm C1 (Fluidigm) using an open app IFC in combination with the PicoPLEX® WGA kit (Rubicon Genomics). The Fluidigm Script Builder software was used to set the cycling parameters for the platform utilizing PicoPLEX chemistry and generate a custom program for use with the C1. The manufacturer’s protocol was used in combination with the protocol generated by the Fluidigm Script Builder software to sort and amplify the single cells. In brief, the IFC was primed with the C1 Harvest reagent, C1 Preloading Reagent and C1 Blocking Reagent. Cells were then prepared in a suspension with the C1 Cell Wash Buffer at a density of 66–333 cells/µl and mixed with the Suspension Reagent in a 3:2 ratio Cell Wash Buffer/Cells and Suspension Reagent. The cells were then loaded into the IFC and their presence confirmed using an Axio Observer Z1 microscope. Lastly the PicoPLEX Pre-Amp and Amplification Reagents were added to the appropriate wells of the IFC. The C1 was run overnight utilizing the thermocycler profile indicated by the PicoPLEX manufacturer. The following day, the amplified material was collected into a 96 well plate with 10 µl of the C1 DNA Dilution Reagent already added in each well. The material could then be stored prior to sequencing long term at −20 °C or used within a week if stored at 4 °C.

### Library preparation and DNA Sequencing

WGA material was first fragmented to an average size of 200 bp using the S2 Focused Ultrasonicator (Covaris, Woburn, USA) following the manufacturer’s instructions (Duty cycle of 10%, Intensity of 5, 200 cycles per burst and Duration 120 sec). 8 ng of WGA product was used as input for fragmentation. Library preparation was performed using the NEBnext Ultra II Kit (New England BioLabs) according to manufacturer’s protocol using the entirety of the amplified material from the C1. Samples were then run through a BioAnalyzer high sensitivity chip (Agilent Technologies, Santa Clara, CA, USA) to control the library’s size distribution and quality. Library quantification was performed using a Sequencing Library qPCR Quantification Kit (Illumina, San Diego, USA). The libraries from the different samples were pooled equimolarly, denatured and diluted to a final loading concentration of 2.5 pM for sequencing. Sequencing was performed on a high throughput Illumina NextSeq. 500 flow cell (Illumina, California, USA) generating 75 bp single reads. Per sample, on average 3.92 ± 1.33 × 10^6^ reads were generated.

### Bioinformatics

Analysis was performed as previously reported^36,37^. Sequencing reads were trimmed using cutadapt^38^ version 1.16 to remove the Illumina adaptor sequences. The trimmed reads were mapped against the Homo sapiens GRCh38 reference genome using Bowtie2^39^ version 2.3.4.1 with local alignment settings. Copy number variation analysis was performed using a previously published^40^. In brief, per bin reads were counted, excluding difficult to sequence regions. After normalization with respect of the GC content and log2 transformation of the read counts per bin, a circular binary segmentation algorithm (DNAcopy, Bioconductor) was applied. The first step in this algorithm was data smoothing with default parameters, followed by the segmentation algorithm with default parameters (min 5 bins in one segment and p-value of 0.05). For each segment, the read counts were replaced by the median of the normalized counts in that segment. The copy number of the sample was determined by minimalizing the sum-of-squares error between the raw copy number and the rounded copy number, calculated for each copy number. The normalized read counts per bin was then multiplied by the copy number, from which a threshold was set based on an internal control to determine copy number. Per sample bin sizes were determined by the clustering of bins at copy integers 1, 2 and 3 with thresholds set to >80%, >62% and >58% for bin sizes of 0.5 Mb, 1 Mb or 2.5 Mb respectively. The threshold for CNV calls was set based on the minimums needed to consistently detect the isochromosome 20; a minimum of 8 consecutive bins at +0.65 for gains at bin sizes of 0.5 Mb and 1 Mb, +0.55 for gains at a bin size of 2.5 Mb, and −0.65 for losses at all bin sizes, with a minimum size of 8 Mb at bins of 0.5 Mb and 1 Mb, and 20 Mb at bins of 2.5 Mb.

## Supplementary information

Supplementary Figure S1

## Data Availability

The datasets generated during and/or analysed during the current study are available from the corresponding author on request.
