# Supplementary material for: Uncovering low-level mosaicism in human embryonic stem cells using high throughput single cell shallow sequencing
Source: Sci Rep. 2019 Oct 16;9:14844. doi: 10.1038/s41598-019-51314-6 (PMC6796059; doi:10.1038/s41598-019-51314-6)
Supplement: Supplementary file 1 — Supplementary Figure S1 [file 41598_2019_51314_MOESM1_ESM.pdf]

Uncovering low-level mosaicism in human embryonic stem cells using high throughput single cell shallow sequencing

Alexander Keller[1], Laurentijn Tilleman[2], Dominika Dziedzicka[1], Filippo Zambelli[3], Karen Sermon[1], Filip Van Nieuwerburgh[2], Claudia Spits[1], Mieke Geens[1]*

1 – Research Group Reproduction and Genetics, Vrije Universiteit Brussel, Laarbeeklaan 103, 1090, Brussels, Belgium 2 – Laboratory of Pharmaceutical Biotechnology, Ghent University, Ottergemsesteenweg 460, 9000, Ghent, Belgium 3 – Clínica EUGIN, Travessera de les Corts 322, Barcelona 08029, Spain

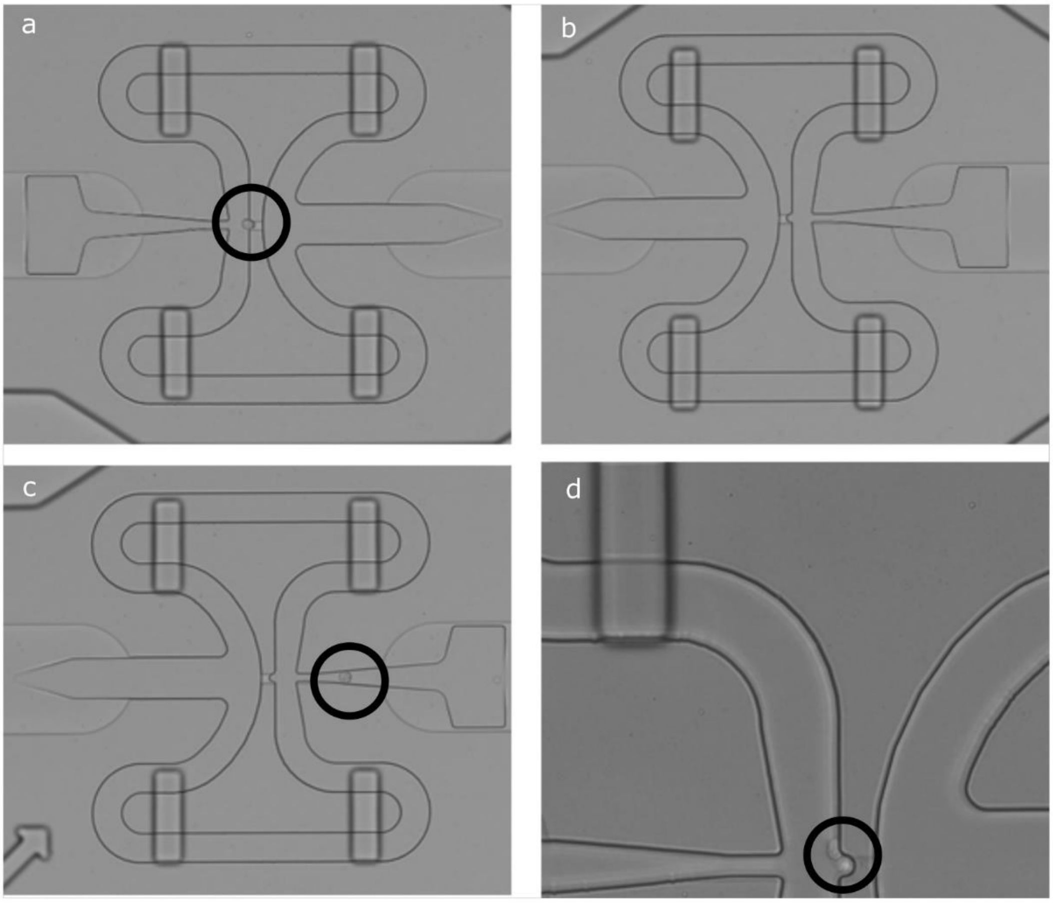

Supplementary Figure 1. Cell capture sites: Images of the C1 chip capture sites. (a) Single cell at the normal capture site. (b) Absence of a cell (c) Single cell in reaction chamber, not on capture site. (d) Two cells at the capture site.
